# Auto-Disinfectant Acrylic Paints Functionalised with Triclosan and Isoborneol—Antibacterial Assessment

**DOI:** 10.3390/polym13132197

**Published:** 2021-07-01

**Authors:** Micaela Machado Querido, Ivo Paulo, Sriram Hariharakrishnan, Daniel Rocha, Cristiana Costa Pereira, Nuno Barbosa, João Moura Bordado, João Paulo Teixeira, Rui Galhano dos Santos

**Affiliations:** 1 Environmental Health Department, National Institute of Health, 4000-055 Porto, Portugal; micaelaquerido@hotmail.com (M.M.Q.); cristiana.pereira@insa.min-saude.pt (C.C.P.); joao.teixeira@insa.min-saude.pt (J.P.T.); 2 EPIUnit, Institute of Public Health, University of Porto, 4099-002 Porto, Portugal; 3 Laboratory for Integrative and Translational Research in Population Health (ITR), 4050-600 Porto, Portugal; 4 Instituto Ciências Biomédicas Abel Salazar, University of Porto, 4099-002 Porto, Portugal; 5 CERENA—Centre for Natural Resources and the Environment, Instituto Superior Técnico, 1049-001 Lisboa, Portugal; ivo.p1691@gmail.com (I.P.); sriram.hariharakrishnan@tecnico.ulisboa.pt (S.H.); jcbordado@ist.utl.pt (J.M.B.); 6 Barbot, 4410-295 Vila Nova de Gaia, Portugal; danielrocha@barbot.pt (D.R.); nunobarbosa@barbot.pt (N.B.)

**Keywords:** auto-disinfectant, acrylic paint, triclosan, isoborneol, functionalised

## Abstract

Environmental surface contamination with microorganisms is a serious concern worldwide. Triclosan and isoborneol present good antimicrobial activity. Their immobilisation to paint substrates allows for development of a material that stays effective over a longer time. In this work, we disclosed the preliminary studies to evaluate the antimicrobial activity of the active molecule after being functionalised with isocyanates for further immobilisation on the paint substrate. Overall, the newly developed non-release antimicrobial coating provides an effective way of preventing the spread of diseases and has been proven to inhibit bacterial growth and with a considerable antimicrobial activity towards *S. aureus*, *E. coli,* and *K. variicola* at the tested concentrations.

## 1. Introduction

Environmental surface contamination with microorganisms is a serious concern worldwide. The presence of fungi, virus, and bacteria in environmental surfaces has been a hot topic of research in the last years and even more recently due to the COVID-19 pandemic.

Several recent studies have proven the frequent contamination of different surfaces with pathogenic microorganisms and the contribution of this to infection spreading, indicating a potential risk and, in many cases, representing a public health issue. These studies have verified environmental contamination in places more prone to microorganisms’ spreading, such as healthcare facilities but also in public spaces such as schools, gyms, hotels, and public transportation [1,2,3,4,5,6].

Many of the cleaning and disinfection protocols often applied are known to be inefficient or insufficient to reduce the microorganisms’ adhesion and growth on surfaces. In some cases, incorrect application of cleaning protocols may cause even more significant problems, contributing to the spread of pathogens from contaminated to clean surfaces [7]. To help solve this global problem, several strategies are being developed by scientists across the world.

Self-disinfecting surfaces are an excellent example of the developed techniques to reduce pathogens transmission to and from environmental surfaces. These surfaces can reduce microorganisms’ survival after close contact, inhibiting the establishment of transmission routes. These surfaces are often coated or impregnated with substances with antimicrobial or biocidal properties [8].

Triclosan is a synthetic, lipid-soluble bisphenol highly used in personal care and hygiene products due to its antimicrobial properties. Triclosan has activity against a broad spectrum of fungi and bacteria, both gram-positive and gram-negative. This substance inhibits bacterial fatty acids synthesis and can disrupt bacterial membranes. Therefore, this chemical is frequently found in the composition of toothpaste, soaps, deodorants, among others, as a preservative [9,10].

Bílek et al. [11] had already evidenced that polyethylene surfaces coated with T-NCO have good antimicrobial activity against *S. aureus* and *E. coli.* Besides, another study regarding T-NCO incorporation on polymeric materials developed by Braid showed that plastic storage boxes impregnated with T-NCO were able to inhibit the growth of *S. aureus, E. coli* and *B. cereus* even though *S. aureus* and *E. coli* presented higher inhibition values (10^4^ and 10^5^ CFU/mL, respectively) comparing to *B. cereus* (10^3^ CFU/mL inhibition) [12].

Ledder et al. also demonstrated that the minimum inhibitory concentration (MIC) of TCS for *B. cereus* was 0.98mg/L, a value superior to the one found for *E. coli* ATCC 25,922 (0.0008 mg/L) in the same study [13]. These results are in accordance with the ones we obtained, with *S. aureus* and *E. coli* being less resistant to TCS than *B. cereus.*

Regarding our results obtained for *E. faecalis*, some previous studies had already evidenced that TCS could not have such a high antimicrobial activity against *E. faecalis* compared to other species we tested.

Perez-Garza et al. [14] developed a study where the hands of volunteers were soiled with *E. coli* or *E. faecalis* at two concentrations 10^3^ CFU/g and 10^6^ CFU/g and then washed with different soaps with antimicrobial properties, one of them containing TCS. This study showed that *E.coli* was not detected in handwashing rinsates (10^3^ CFU/g) containing TCS or decreased its levels over time (10^6^ CFU/g) while *E. faecalis* was detected for both concentrations (10^3^ CFU/g and 10^6^ CFU/g) and maintained its levels during a 20h period, suggesting that *E. faecalis* was more resistant to the presence of TCS comparing to *E.coli* [14].

Furthermore, Jones et al. [15] have shown that the MIC of TCS for *E. faecalis* strains (7 mg/L) is much higher than for *S. aureus* (0.1-0.6 mg/L) or *E. coli* (0.2–0.3 mg/L) strains. No information was found regarding the MIC of TCS for *K. variicola*. However, in this same study, Jones et al. [15] demonstrated that the MIC for *K. pneumoniae* (0.3–0.8 mg/L) (*K. variicola* belongs to the *K. pneumoniae* complex) [16] was similar to those found for *S. aureus* and *E. coli*, and our results proved similar values of antimicrobial activity (R) of TCS for the three species [15].

Isoborneol is a monoterpene found in the constitution of several plants and essential oils. Being a component of several essential oils, it has been used as a perfume since it releases a camphor/woody fragrance. Isoborneol is also commonly used as a food additive due to its characteristic fruity and spicy flavour added to baked goods such as candies, cakes, or beverages. This substance has been studied as a potential virucidal agent against herpes simplex virus type 1 [17] and for its potential antifungal and antimicrobial activity, namely against *Staphylococcus aureus* [18].

Although ISB is frequently found on essential oils of several plants and some studies evaluate the antimicrobial properties of those oils, there is very little information regarding the antimicrobial activity of ISB as an isolated component.

In a study developed by Zhu et al. [18], I-NCO was found to be one of the main constituents of *Curcuma wenyujin* essential oil. In this study, isolated ISB proved to have strong inhibitory activity against *S. aureus*, presenting the largest inhibition zone diameter on a test involving several components of this essential oil. In addition, ISB also presented the lowest MIC for *S. aureus* (62.5 µg/mL) compared to the other *Curcuma wenyujin* essential oil components. No more additional studies investigating the antimicrobial properties of ISB alone were found. We also did not find MIC values of ISB for the other bacterial species used in our study. However, some papers demonstrate the antimicrobial properties against different bacteria of essential oils having high percentages of ISB on their constitution [19,20].

Previous work by Silva et al. (2019) [21] disclosed a straightforward process for active principle immobilisation. The process is based on isocyanates aimed at non-biocide-release coatings. In Silva et al. (2019) [21] work, it was demonstrated that with this procedure, an active molecule once could be immobilised towards a covalent bond upon urethane formation between the reactive groups from the active molecule and the isocyanate. Afterwards, the functionalised moiety can be grafted/bonded to the paint chemical structure. Furthermore, it was demonstrated that functional reactive biocides, which can remain immobilised through covalent bonds to polymeric materials, can extend the effectiveness of the surfaces, avoiding the loss of efficiency, when compared with the conventional releasing strategies [21].

**Figure 1:**
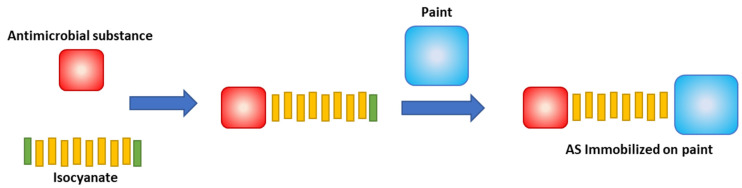
Immobilisation process for antibacterial molecules in the polymeric matrices with –OH function.

The preliminary work presented herein evaluates the antibacterial activity of wall paint after the immobilization of the active principles, via polyurethane, (Figure 1). The main goal is to obtain a self-disinfecting paint with application on healthcare facilities, public spaces, and other contamination-prone environments. Triclosan (TCS) and Isoborneol (ISB) were incorporated in water-based commercial acrylic paint by covalent immobilisation. To test for their antibacterial efficiency, the paint samples were challenged with five different bacteria that are frequently associated with healthcare-acquired infections and/or environmental contamination—*Staphylococcus aureus*, *Escherichia coli, Bacillus cereus, Enterococcus faecalis* and *Klebsiella variicola*. The antibacterial activity was assessed based on the methodologies described in the standards ISO 222,196 [22] and JIS Z2801 [23].

## 2. Materials and Methods

### 2.1. Antimicrobial Paints Preparation

Triclosan [5-chloro-2-(2,4-dichlorophenoxy)phenol], 99%, provided by Alfa Aesar, and Isoborneol [(1R,2R,4R)-1,7,7-trimethylbicyclo [2.2.1]heptan-2-ol], 93%, provided by Acros Organics, were the selected antibacterial substances to be immobilized. Isophorone diisocyanate, a mixture of cis- and trans-isomers for synthesis, was purchased from Sigma-Aldrich.

The covalent immobilisation through urethane link formation of the antibacterial substances in polymeric matrices was performed based on the method by Silva et al. (2016) [24].

The first step consists of the functionalisation of the antibacterial substances with a reactive diisocyanate to allow further immobilisation through covalent bonds onto compatible polymeric matrices backbone.

The functionalisation step starts with the dissolution of each antibacterial substance in a suitable solvent to further immobilization in polymeric coating formulation. Tetrahydrofuran (THF) was the selected solvent for both active principles. The isoborneol and troclosan solutions were added dropwise into a three-necked round bottom flask containing IPDI, the reaction proceeds at stoichiometric and equimolar conditions at room temperature, under mechanical agitation (200–300 rpm) and inert atmosphere conditions, for 24 h. Afterwards, the reactional mixture was filtrated and concentrated under vacuum, in a Butchi R-210/215 rotovapor, to remove the solvent. The obtained functionalised antibacterial substances were named T-NCO (Triclosan-N=C=O) and I-NCO (Isoborneol-N=C=O).

The substances T-NCO and I-NCO were then chemically immobilised in the acrylic paint. The functionalised antibacterial substances were added to the paints matrix as additives. The mixing was performed in a mechanical stirrer with shear force at 800 rpm speed, for 5 min at normal temperature and humidity conditions (25 °C, 50% HR). The substances were mixed at three different concentrations each: T-NCO was incorporated at 0.0006 g/L, 0.0009 g/L, 0.0012 g/L and I-NCO at 0,6 g/L, 0.9 g/L, 1.2g/L. T-NCO or I-NCO paints were applied on PVC coupons (50 mm × 50 mm), forming a layer of 200 µm of thickness. The drying time of the paint after application was 24 h.

### 2.2. Functionalised Antibacterial Molecules

Fourier Transform Infrared Spectroscopy (FTIR) analysis in PerkinElmer FTIR Spectrometer coupled to an attenuated total reflectance (ATR) unit from PerkinElmer with an individual Diamond crystal was performed with both antibacterial substances and their functional counterparts. Studies were carried out in a frequency range of 650–4000 cm^−1^ with 4 cm^−1^ resolution. FTIR-ATR also evaluated the reaction progress.

### 2.3. Bacteria and Growth Conditions

For the antibacterial activity testing of the paints, two gram-negative and three gram-positive bacteria were selected—the gram-negative species *Escherichia coli* (NCTC 25922) and *Klebsiella variicola* (ATCC 31488), and the gram-positive species *Staphylococcus aureus* (ATCC 25923), *Bacillus cereus* (clinical isolate) and *Enterococcus faecalis* (NCTC 775).

Bacteria were grown on tryptic soy agar (TSA) (VWR, Radnor, PA, USA) plates overnight at 37 °C. The bacterial inoculum was prepared in maximum recovery diluent (MRD) (VWR, Radnor, PA, USA), adjusting to a cell density of approximately 6 × 10^5^ colony-forming units per millilitre (CFUs/mL).

### 2.4. Antibacterial Activity

The antibacterial activity was assessed following ISO 222,196 [22] and JIS Z2801, with minor modifications [23].

The PVC coupons (50 mm × 50 mm) samples containing T-NCO or I-NCO were sterilised using germicidal UV light (254 nm; Fischer Scientific, Porto Salvo, Portugal) for 15 min on each side. Each sample was placed on a sterile petri dish and inoculated with 400uL of the prepared bacterial inoculum. The samples were covered with previously sterilised parafilm (40 × 40 mm) and incubated for 24 h at 37 °C with high humidity levels. At time zero control, a set of identical samples were immediately processed without incubation, as follows.

After 24 h incubation, 10 mL of tryptic soy broth with neutralising agents—Tween 80, lecithin, histidine and sodium thiosulfate (TSB THLth, VWR, Radnor, PA, USA) were added to each petri dish, the parafilm removed, and the petri dish was gently stirred. Dilutions (from 10^−1^ to 10^−5^) of this solution were made using MRD and placed on sterile Petri dishes (*n* = 2 replicates were performed). Following, 15 mL of previously melted plate count agar (PCA) (VWR, Radnor, PA, USA) was added to each petri dish. After drying, the plates were incubated for 48 h at 37 °C with high humidity levels. After this incubation step, the CFUs on each plate were enumerated, and the number of viable bacteria per cm^2^ per sample was determined.

According to ISO 22,196 [22] and JIS Z 2801 15], the antibacterial activity index (R) is obtained following the equation:

(1) $$R=\left(U_{t}-U_{0}\right)-\left(A_{t}-U_{0}\right)=U_{t}-A_{t}$$

where *U*_0_ is the average of the common logarithm of the number of viable bacteria, in CFUs/cm^2^, recovered from the control paint samples immediately after inoculation (T_0_); *U*_t_ is the average of the common logarithm of the number of viable bacteria, in CFUs/cm^2^, recovered from the control paint samples after 24 h (T24); *A*_t_ is the average of the common logarithm of the number of viable bacteria, in CFUs/cm^2^, recovered from the antibacterial paint samples after 24 h (T24).

## 3. Results and Discussion

### 3.1. Isocyanate Functional Antibacterial Substances

**Figure 2:**
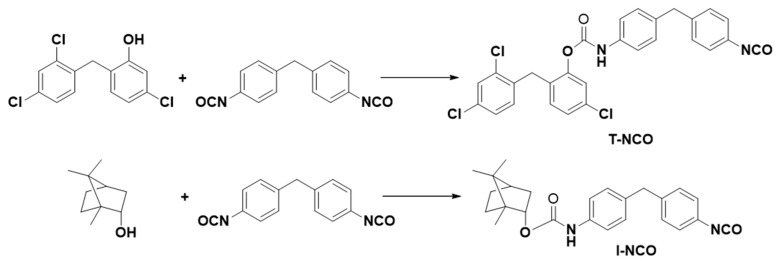
Chemical scheme of the isocyanate functionalisation of the antibacterial molecules.

The commercial antibacterial substances were functionalised with an isocyanate function. Conversions as high as 95% ± 5% were obtained. The general reaction between triclosan and isoborneol with isocyanate was expected to proceed according to Figure 2. FTIR-ATR confirmed the immobilisation of triclosan and isoborneol. Such immobilisation occurred by forming a urethane group between the OH of both antibacterial molecules and one of the isocyanate groups.

**Figure 3:**
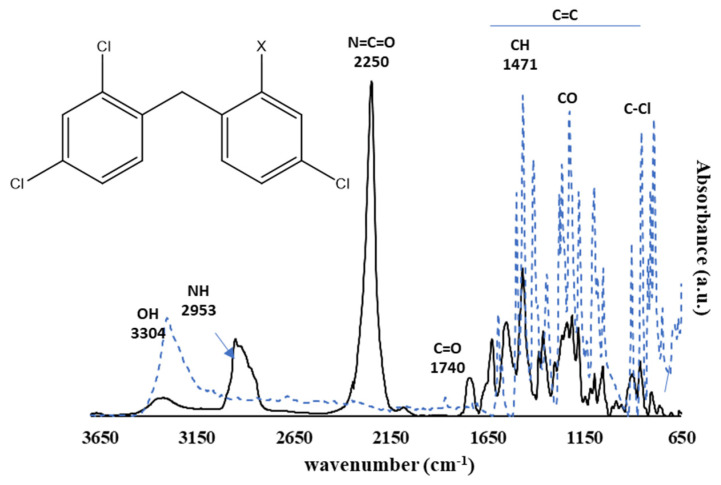
Normalised infrared spectra (FTIR-ATR) obtained from triclosan (dashed-line, X = OH) and its isocyanate functional derivative (solid line, X = OCONH–R–NCO, T–NCO).

**Figure 4:**
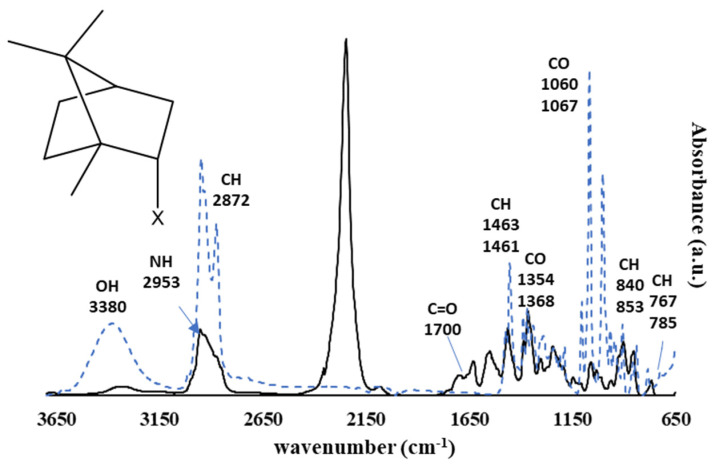
Normalised infrared spectra (FTIR-ATR) obtained from isoborneol (dashed-line, X = OH) and its isocyanate functional derivative (solid line, X = OCONH–R–NCO, I–NCO).

Figure 3 shows the spectra of triclosan and its isocyanate functional derivate (T-NCO), and Figure 4 shows the spectra of isoborneol and its isocyanate functional derivative (I-NCO).

From Figure 3, the main characteristic groups of triclosan can be observed, and the isocyanate functionalised triclosan.

The observed spectrum range between 3378 cm^−1^ and 3128 cm^−1^ is assigned to the OH group, while the peaks in the range between 1600 cm^−1^ and 1030 cm^−1^ are associated with the C=C of the aromatic backbone. The range between 914 cm^−1^ and 740 cm^−1^ is assigned to the C–Cl stretch [21].

For the functional Triclosan (T-NCO), new bands appeared, such as the characteristic band located at 2250 cm^−1^ and assigned to the N=C=O isocyanate stretch, which confirmed the functionalisation effectiveness. Besides, the functionalisation is confirmed by the appearance of the assigned C=O stretch band located at 1740 cm^−1^, attributed to unsaturated ketones, and corroborating the urea bond formation between the isocyanate function and the OH group from triclosan. Furthermore, the consumption of the OH group band and the appearance of a peak at 2953 cm^−1^ associated with a secondary amine stretch is also observable.

Figure 2 shows the main characteristic groups of isoborneol, as well as the isocyanate functionalised isoborneol.

Isoborneol (Figure 4) presents a spectrum range between 3648 cm^−1^ and 3188 cm^−1^, assigned to the OH group, the range between 2998 cm^−1^ and 2826 cm^−1^ is assigned to the C-H aliphatic ring, as the peaks at 840 cm^−1^ and 785 cm^−1^.

The functional Isoborneol (I-NCO) (Figure 4) also presents the intense band located at 2247 cm^−1^, assigned to the N=C=O isocyanate stretch, confirming the presence of the isocyanate functionalisation. Additionally, the disappearance of the OH band indicates that the hydroxyl reacted with the isocyanate to afford the reactive polyurethane. It also presents the appearance of the assigned C=O stretch band located at ~1700 cm^−1^, attributed to the polyurethane resulting from the reaction of the isocyanate and the OH group from isoborneol. Additionally, there was the appearance of a peak at 2953 cm^−1^ associated with a secondary amine stretch.

According to Barbot’s commercial standards, the paint samples with functionalised T-NCO and I-NCO were subjected to standard testing procedures to ensure the right viscosity, density, and opacity for application.

### 3.2. Antibacterial Activity

The paint samples were considered to have antibacterial activity when presenting a value of R equal or superior to two, according to JIS Z 2801 recommendations [23].

**Figure 5:**
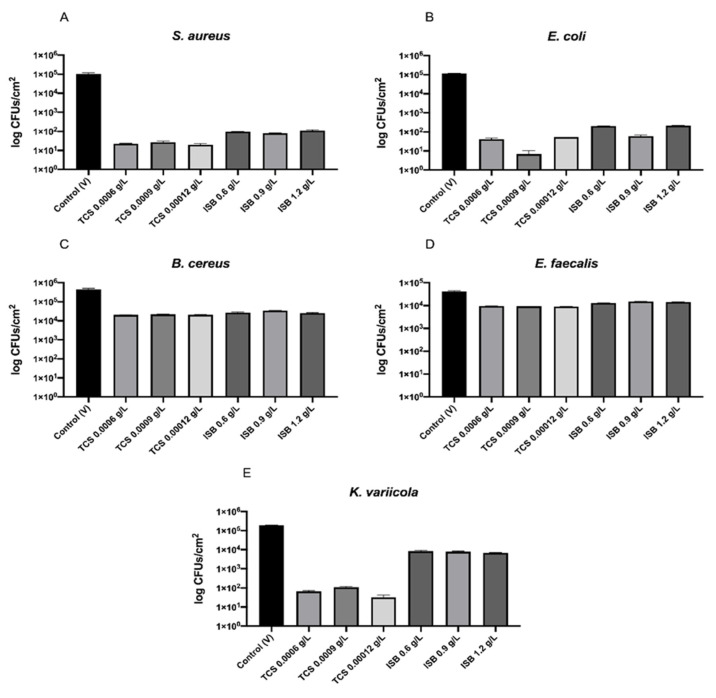
Average number of viable CFUs/cm2 of (**A**) *S. aureus*, (**B**) *E. coli*, (**C**) *B. cereus*, (**D**) *E. faecalis* and (**E**) *K. variicola* recovered from each paint sample after 24 h of contact. The values are presented on a logarithmic scale.

The three paints containing different concentrations of T-NCO were able to reduce the number of bacteria comparing to the control during the 24 h period of incubation (Figure 5). In addition, the paints with T-NCO presented antibacterial activity (R ≥ 2) against S. aureus, *E. coli* and *K. variicola*, as exhibited in Table 1. However, these paints did not show antibacterial activity (R < 2) against B. cereus and *E. faecalis*.

Regarding the paints containing I-NCO, they proved their antibacterial activity against S. aureus and E. coli. However, these paints did not show antibacterial activity against *B. cereus*, *E. faecalis* and *K. variicola*. Nevertheless, they were able to reduce the number of bacteria after 24 h of contact, comparing to the control for all bacterial species.

The three paints containing T-NCO were able to reduce the number of viable bacteria after 24 h of contact for all tested bacterial species, comparing to the control. No apparent differences were detected between different concentrations of T-NCO for any bacteria.

The paints with T-NCO presented antibacterial activity against *S. aureus*, *E. coli* and *K. variicola* with identical results for the three bacterial species, with R values varying between 3.2 and 4.2. Nonetheless, these paints did not show antibacterial activity against *B. cereus* and *E. faecalis.* For *B. cereus,* the T-NCO paints presented a reduction of around 1 log, and for *E. faecalis,* the value was lower ~0.6 log.

Respecting the paints containing ISB, the three tested concentrations could inhibit the bacterial growth during the 24 h of incubation period at some level. Once more, no apparent differences were detected between the different concentration of ISB in the paints. These paints presented antibacterial activity against *S. aureus* and *E. coli*. However, they did not exhibit activity against *B. cereus*, *E. faecalis,* and *K. variicola.*

It is essential to point out that, despite the results of antibacterial activity obtained, all paints, either containing TCS or ISB, were able to reduce the number of viable bacteria after 24 h of contact, compared to the control.

The majority of paints have in their constitution biocidal substances used as preservatives, to prevent the growth of microorganisms in storage conditions. However, the addition of substances with antibacterial properties confer the paints an efficient and fast response to the presence and potential development of microorganisms to the surfaces where the paints are applied. [1] The immobilization of different types of substances with antibacterial properties on paints has been successful, namely on acrylic water-based paints [2].

The capacity of the modified paint to have a better performance on microorganisms elimination comparing to the regular paint, is closely related to the biocide properties of immobilized substances, in our case, TCS and ISB.

As previously said, TCS interacts with bacterial membranes disrupting them and inhibiting bacterial fatty acids synthesis. There is very little information available about ISB mechanism of action against microorganisms as an isolated substance. However, ISB is frequently a major component of essential oils that have the ability to increase apoptosis on bacteria and to disrupt cell wall and membrane [3,4].

## 4. Conclusions

In this work, commercial substances with described antibacterial proprieties, such as Triclosan and Isoborneol, were immobilised in polymeric coatings by a recently developed immobilisation process without losing their antibacterial activity. This process was proven to be capable of producing an active paint that inhibits bacterial growth and a considerable antibacterial activity towards *S. aureus*, *E. coli,* and *K. variicola* at the tested concentrations.

Overall, the newly developed non-release antibacterial coating provides an effective way of preventing the spread of diseases. In addition, by being immobilised by a chemical bond, the migration or transmission of the antibacterial substances will gradually be reduced. Thus, toxicity risks to the people who touch the coated surfaces will be mitigated.

This type of coating highly reduces the possibility of spreading the pathogens, allowing control over community infections and improving public health.

The need for functional strategies promoting the disinfection of environments prone to microbial growth is in high demand. Moreover, the world is still struggling with a pandemic. The described methodology is an efficient path to obtain improved and safer environments.

## Figures and Tables

**Table 1:** Antibacterial activity (R) values obtained for each bacterial species after contact with the different paint samples.

	*S. aureus*	*E. coli*	*B. cereus*	*E. faecalis*	*K. variicola*
**TCS 0.0006 g/L**	3.7	3.4	1.3	0.6	3.5
**TCS 0.0009 g/L**	3.6	4.2	1.3	0.6	3.2
**TCS 0.0012 g/L**	3.7	3.3	1.3	0.7	3.8
**ISB 0.6 g/L**	3.0	2.7	1.2	0.5	1.4
**ISB 0.9 g/L**	3.1	3.3	1.1	0.4	1.4
**ISB 1.2 g/L**	3.0	2.7	1.2	0.5	1.5

## Data Availability

The data presented in this study are available on request from the corresponding author.
